# The Challenges of Diagnosing, Managing, and Preventing Pediatric Delirium

**DOI:** 10.3390/children12070918

**Published:** 2025-07-11

**Authors:** Juliana Patrícia Chaves de Almeida, Yu Kawai, Arnaldo Prata-Barbosa, Roberta Esteves Vieira de Castro

**Affiliations:** 1 Pediatric Intensive Care Unit, Department of Pediatrics, Federal Hospital of Lagoa, Rio de Janeiro 22470-050, Brazil; juliana.almeida@souzamarques.br; 2 Department of Pediatrics, Souza Marques School of Medicine, Rio de Janeiro 21310-310, Brazil; 3 Department of Pediatrics, Division of Pediatric Critical Care Medicine, Mayo Clinic Children’s, Rochester, MN 55905, USA; kawai.yu@mayo.edu; 4 Department of Pediatrics, D’Or Institute of Teaching and Research (IDOR), Rio de Janeiro 22281-100, Brazil; arnaldo.prata@idor.org; 5 Department of Pediatrics, Pedro Ernesto University Hospital, Rio de Janeiro State University (UERJ), Rio de Janeiro 20550-013, Brazil

**Keywords:** delirium, pediatric delirium, diagnosis, pediatric intensive care unit, prevention, management

## Abstract

Pediatric delirium (PD) is an acute neuropsychiatric syndrome marked by fluctuating disturbances in attention and cognition, frequently observed in pediatric intensive care units (PICUs) and associated with increased morbidity, mortality, and long-term cognitive impairment. Despite its clinical significance, PD remains underdiagnosed due to challenges inherent in assessing consciousness and cognition in children at varying developmental stages. Several bedside tools have been developed and validated in recent years, including the Cornell Assessment of Pediatric Delirium (CAPD), PreSchool Confusion Assessment Method for the Intensive Care Unit (psCAM-ICU); Pediatric Confusion Assessment Method for the Intensive Care Unit (pCAM-ICU), and Sophia Observation Withdrawal Symptoms—Pediatric Delirium Scale (SOS-PD), enhancing early recognition and management of PD in critically ill children. This narrative review explores the historical background, epidemiology, risk factors, pathophysiology, clinical subtypes, diagnostic tools, and current prevention and treatment strategies for PD from newborns to 21 years old. The screening tools available and the integration of non-pharmacological interventions, such as environmental modifications and family-centered care, as well as cautious and selective pharmacological management, are emphasized in this review. Early identification and targeted interventions are essential to mitigate the adverse outcomes associated with PD.

## 1. Introduction

Delirium is a potentially life-threatening manifestation of acute brain dysfunction marked by fluctuating and transient alterations of consciousness and characterized by a wide variety of clinical manifestations. It designates an acute organic failure associated with a poor prognosis [1,2]. An episode of delirium is triggered by an acute clinical event related to the disease and/or its treatment and should be considered a medical emergency [1].

The concept of delirium has its roots in ancient medicine. Hippocrates described mental confusion in patients with fever or serious illnesses, recognizing their transitory nature. In the 1st century AD, Aulus Cornelius Celsus used the term “delirium”, literally “to leave the furrow while plowing,” to define a “departure from reason,” consolidating the origin of the term [3]. In the early 20th century, the Swiss psychiatrist, Eugen Bleuler, also addressed delirium, but with an erroneous view in the pediatric context: he stated that delirium was so common in children that it did not deserve greater diagnostic attention, reflecting the thinking of the time, which underestimated the importance of acute mental suffering in children [4,5]. Contrary to this viewpoint, Leo Kanner, the founder of modern child psychiatry, recognized the clinical relevance of acute mental state changes in hospitalized children. His more nuanced understanding of child psychopathology laid the foundation for pediatric delirium (PD) to be recognized decades later as a severe disorder with relevant prognostic implications in pediatric intensive care units (PICUs) [5,6].

The recognition of PD in neonatal and pediatric patients up to 21 years of age has advanced significantly in recent years, and its assessment has been possible thanks to the development of reliable, valid, and easy-to-use diagnostic tools by a non-psychiatric professional at the bedside [2,7]. These instruments have enabled more accurate epidemiological assessments of delirium in PICUs [2]. In PICUs, delirium is a serious complication associated with prolonged hospitalizations, increased risk of infections, increased need for mechanical ventilation (MV), increased mortality, increased morbidity, and higher costs [8,9,10,11]. Moreover, delirium occurrence during PICU stay is associated with worsening health-related quality of life (HRQoL) in children from the time of admission to the follow-up period after discharge [12] as well as post-intensive care syndrome (PICS) [13].

Considering the numerous potential complications and the fact that delirium represents a clinical manifestation of acute brain dysfunction, it is regarded as far more than a mere behavioral disturbance. The presence of delirium indicates a pathological process warranting a thorough investigation of contributing risk factors. For this reason, delirium has increasingly been recognized by researchers worldwide as a “new vital sign” in critically ill patients [14].

Given its clinical impact, this review highlights current developments regarding delirium in pediatrics, summarizing key points of its epidemiology, risk factors, pathophysiology, and clinical manifestations. It will also focus on screening tools and non-pharmacological and pharmacological approaches to prevention and treatment.

For this narrative review, the authors conducted a comprehensive literature search of peer-reviewed articles on PD, covering its epidemiology, risk factors, pathophysiology, diagnosis, prevention, and management. Sources were identified through searches in PubMed and Google Scholar, using keywords, such as “ delirium”, “children”, “pediatric delirium”, “intensive care”, “diagnosis”, “management”, “prevention”, “treatment”, and related terms. Additionally, key reference texts and recent international guidelines were reviewed. No formal date or language restrictions were applied; however, emphasis was given to the literature published within the past ten years. The selection prioritized studies with validated methodologies and clinical relevance, as well as those addressing recent advances or knowledge gaps. Bibliographic cross-referencing was performed to ensure the inclusion of seminal and highly cited works. The final references reflect both a broad historical perspective and the most up-to-date scientific understanding of PD.

## 2. Epidemiology

Epidemiological research on delirium produces reliable estimates when it applies standardized diagnostic criteria or validated screening instruments and uses samples that adequately reflect the target population [15,16]. Recent studies show that delirium prevalence in critically ill children varies widely according to the population studied and the diagnostic tools applied [2,5], ranging from 7 to 65.9% [17,18,19]. In 2017, a point prevalence study that included 835 pediatric patients in 25 PICUs from five countries showed a prevalence of 25% for PD. This prevalence was higher (38%) for children in the PICU for six or more days [20]. Another multicenter study that included 27 cardiac PICUs in North America found that the one-day point prevalence of delirium in pediatric patients in the postoperative period of cardiac surgery performed in the last 30 days was 40% [21]. More recently, a systematic literature review showed that the pooled prevalence of delirium is up to 34% of patients admitted to PICUs [19]. In Neonatal Intensive Care Units (NICUs), a recent study estimated the prevalence of delirium at 22% [22].

The incidence can also vary depending on the location and significant comorbidities. In a Brazilian PICU, the incidence was 7.7% [23], while in the United States, it was as high as 72.8%, as reported by Difabio et al., around the same timeframe [24]. In a cohort of eight children who required venoarterial extracorporeal membrane oxygenation, delirium occurred in all of them over 8 months [25]. More recently, Gray et al. published a retrospective analysis of a cohort of 149 children with Coronavirus disease 2019, and delirium was diagnosed in 29% of patients [26].

The most common framework categorizes delirium according to psychomotor behavior, distinguishing it as hypoactive (such as drowsiness or lethargy), hyperactive (such as agitation or restlessness), or mixed [27]. It is interesting to highlight that, regarding the motor subtype, hypoactive delirium is the most prevalent in children, as well as in adults [28,29,30], which can reach >60% in PICUs [2,29]. Despite being more easily recognized, the hyperactive subtype is the least prevalent, and its occurrence may be less than 10% [2,29].

## 3. Risk Factors

Delirium risk factors are divided into two large groups: predisposing and precipitating. Predisposing risk factors are inherent to the patient, demonstrating their vulnerability to delirium and are not modifiable. In pediatric patients, the predisposing risk factors most described in the literature include developmental delay; age less than 2 years; female sex; pre-existing medical condition (mainly cyanotic heart disease); high severity of illness, evidenced by high scores on the PRISM (Pediatric Risk of Mortality) and PIM (Pediatric Index of Mortality) scores, and poor nutritional status (evidenced by low albumin levels) [9,21,31,32,33].

Precipitating risk factors are considered triggers for the development of delirium and are related to the disease, the treatment, and the environment where the patient resides. The PICU is extremely deliriogenic due to the necessary critical care delivered, the iatrogenicity that accompanies it, as well as the inherent nature of the environment [33]. These risks are considered modifiable and require close attention to mitigate the risks of developing PD [1,16,34]. There are numerous known independent risk factors, such as the use of MV and physical restraint, admission diagnosis related to a neurological disorder, sleep deprivation, and liver failure/hepatic dysfunction [10]. Other factors that trigger delirium include coma and deep sedation; length of stay in the PICU; red blood cell transfusion; prolonged cardiopulmonary bypass time; immobilization, as well as changes in sodium and potassium levels [21,31,32,35,36]. Exposures to antiemetics, antiepileptics, corticosteroids, inotropes, opioids, benzodiazepines, dexmedetomidine, and vasoactive medications are also mentioned in the literature as deliriogenic [9,21,31,35]. Finally, the use of anticholinergic medications can culminate in anticholinergic burden, a phenomenon recognized as an essential risk factor for delirium in adult patients and which also appears to be present in pediatrics [36].

In 2023, Ista et al. conducted a systematic review with meta-analysis on non-modifiable and potentially modifiable factors associated with delirium in hospitalized children. Seven factors associated with greater odds of developing delirium during pediatric critical illness were identified, with statistical significance: developmental delay, need for MV, use of physical restraints, and receipt of either benzodiazepines, opiates, steroids, or vasoactive medication. However, contrary to what some studies have shown, no associations were found with age under two years and the use of anticholinergics. Therefore, further studies are needed to identify PD risk factors [36,37].

Concerning the ICU environment, it is well-studied that the ICU has significant noise and light pollution. The World Health Organization (WHO) recommends that inpatient hospital settings not exceed 40 decibels (dB) [38]. However, Kawai et al. showed that the WHO goal was not met even in unoccupied bed spaces [38]. Another study illustrated objective evidence of noise and light pollution in the PICU [39]. These concerns may be associated with poor sleep quality and potentially increase the risk of developing PD [32,38,39,40]. Finally, the unfamiliar aspect of the PICU environment can add to the iatrogenic risks, especially in the pediatric population and developmentally delayed children [41,42,43,44]. One study showed that the lack of family member presence in the ICU may be associated with increased risk of developing PD [45].

## 4. Pathophysiology

The pathophysiology of delirium is not well understood, much less in children. In adults, delirium may arise from the complex interplay between predisposing vulnerabilities, such as aging or neurodegeneration, and acute precipitating insults, including infection, surgery, or metabolic disturbances. Its multifactorial pathophysiology involves neuroinflammation, impaired brain energy metabolism, blood–brain barrier dysfunction, and widespread neurotransmitter imbalances. These factors contribute to neuronal dysfunction, altered connectivity of brain networks, and failure of the ascending arousal system, ultimately leading to the hallmark clinical features of delirium (disrupted attention, cognition, and arousal). Although individual mechanisms may vary by etiology, they converge to impair functional brain integration, particularly in individuals with compromised cognitive reserves [16].

Delirium is conceptualized as an acute brain failure resulting from the breakdown of systems integration within the central nervous system (CNS). The “systems integration failure hypothesis” posits that delirium arises from the dynamic interplay among multiple pathophysiological mechanisms, including neuroinflammation, oxidative stress, neuronal aging, circadian rhythm dysregulation, neurotransmitter imbalances, and impaired sensory processing. These disturbances impair neural connectivity and disrupt cognitive integration, leading to the core clinical domains of delirium: cognitive and attentional deficits, emotional and circadian dysregulation, and altered psychomotor behavior. The severity and presentation depend on individual vulnerabilities (substrates) and precipitating insults, producing heterogeneous phenotypes across patients [46].

In addition to neuronal immaturity in the pediatric age group [20], Traube et al. observed that the highest rates of delirium were among critically ill children admitted due to infectious or inflammatory conditions in an international point-prevalence study. This finding supports the theory that inflammation may be a central factor in PD development. According to the neuro-inflammatory hypothesis, systemic inflammation triggers cytokine release, which in turn affects the CNS in unclear ways, leading to synaptic and neuronal disturbances and, eventually, to the clinical manifestations of delirium. Although adult studies have documented elevated levels of pro-inflammatory cytokines in patients with delirium, these observational findings have not established a direct causal link. It is also plausible that the observed association may reflect impaired perfusion rather than inflammation, as some of these children may have experienced episodes of reduced end-organ blood flow during their PICU stay. Therefore, the authors agree that further research is needed to clarify the role of the immune response in delirium pathogenesis, particularly in pediatric populations [24].

## 5. Clinical Manifestations

According to the Diagnostic and Statistical Manual of Mental Disorders criteria, fifth edition text-revised (DSM-5-TR) of the American Psychiatric Association (APA), delirium is characterized by an acute disturbance of attention and consciousness, with sudden onset (within hours or days) and fluctuating course, accompanied by cognitive changes such as disorientation, impaired memory or changes in language and perception, not explained by another neurocognitive disorder or resulting from a medical condition, intoxication, withdrawal or multiple physiological factors [47]. Although frequently occurring in patients with delirium, the presence of auditory and/or visual hallucinations is not necessary for diagnosis [47,48,49,50].

Considering the diverse manifestations of delirium, optimal treatment approaches should be directed toward the underlying mechanistic subtype. Nonetheless, prior efforts to characterize this heterogeneity have primarily focused on classifications derived from symptoms or risk factors. The most widely used classification system defines delirium based on psychomotor activity, identifying it as hypoactive, hyperactive, or mixed [27].

## 6. Diagnosis

At the time of writing, the diagnosis of delirium can only be made by clinical assessment [51]. Unlike other organ dysfunctions, there are no laboratory or imaging studies with adequate accuracy to diagnose delirium. For instance, acute kidney injury can be diagnosed with creatinine or the degree of urine output [52], which is easy to measure. CNS organ failure, on the other hand, requires detailed symptomatic assessment and can be challenging in critically ill children, mainly because pediatric patients have different cognitive development levels and limited communication ability that can be exacerbated in acute illness [5,50]. The gold standard for diagnosis consists of psychiatric evaluation using the DSM-5-TR [47]. However, the availability of psychiatrists in PICUs is quite scarce. Furthermore, there is a need for frequent and timely evaluations to optimize the diagnosis and management of delirium. Thus, using reliable and valid tools that are quick and easy to use to assess the primary components of delirium by the PICU team in the absence of a psychiatrist is justified [2]. Some tools for the PD screening have been described in the literature and summarized below.

### 6.1. Delirium Rating Scale and Delirium Rating Scale-Revised-98

The Delirium Rating Scale (DRS-88), initially developed for adults, was retrospectively evaluated for pediatric use by Turkel et al. in 2003 in a study with 84 patients aged six months to 19 years [53]. Comprising ten items that assess symptom onset and core features of delirium, the tool yields a maximum score of 32, with scores ≥ 13 indicating delirium. However, its diagnostic performance could not be fully validated due to the study’s retrospective design [53,54]. The Delirium Rating Scale-Revised-98 (DRS-R-98), a revised version of the DRS-88, includes 16 items (13 assessing delirium severity and three for diagnosis), each scored from 0 to 3. A score above 15 on the 18-point severity scale supports a delirium diagnosis. The tool also classifies motor subtypes based on items evaluating increased or reduced activity within the previous 24 h. The original English version demonstrates high sensitivity, specificity, inter-rater reliability, and strong validity compared to the original DRS-88 [55,56]. Although both tools demonstrate strong psychometric properties in adults, a prospective study by Janssen et al. found that their applicability in the PICU is limited, with DRS-88 usable in 66.9% and DRS-R-98 in only 46.8% of cases, mainly due to challenges in assessing cognitive domains in younger children [57].

### 6.2. Pediatric Anesthesia Emergence Delirium Scale

The Pediatric Anesthesia Emergence Delirium Scale (PAED), the first instrument designed to assess delirium in children, comprises five items scored by severity, with the total score reflecting the likelihood of delirium. Initially developed for detecting emergence delirium in children aged 18 months to 6 years following anesthesia, it showed moderate sensitivity (64%) and high inter-rater reliability (0.84) [58]. In a 2011 prospective study, Janssen et al. validated the scale for use in PICU patients aged 1 to 17 years, demonstrating excellent psychometric performance, including high sensitivity (91%), specificity (98%), and internal consistency (Cronbach’s alpha 0.89), with a recommended cutoff score of ≥8 [57]. A post hoc analysis by Blankespoor et al. supported a simplified three-point severity scoring system, preserving strong diagnostic accuracy (Receiver Operating Characteristic 0.98, sensitivity 100%, specificity 96.7%). Nonetheless, further prospective research was advised to confirm its broader clinical utility [59]. A German version of the PAED scale was applied to 64 patients in a tertiary PICU in Germany. Initial assessments showed a sensitivity of 69.2%, while repeated measurements revealed a reduced sensitivity of 35.9%. Regarding specificity, the tool demonstrated 98.0% for initial assessments and 99.4% for repeated evaluations [60]. Strengths of the PAED include its focus on observing neurobehavioral symptoms, which makes it suitable for evaluating younger children and potentially adults with developmental delay, in whom neurocognitive symptoms are difficult or impossible to assess. However, the validity and generalizability of the PAED for the diagnosis of all delirium subtypes in PICUs are uncertain, being a tool limited to the less frequent hyperactive subtype [2,30,57,61,62].

### 6.3. Cornell Assessment of Pediatric Delirium

In 2012, Silver et al. introduced the Cornell Assessment of Pediatric Delirium (CAP-D), a modification of the PAED that captures hypoactive and mixed delirium. In a pilot study with 50 PICU patients aged 3 months to 21 years, the CAP-D showed excellent agreement with DSM-IV (κ = 1.0), with overall sensitivity and specificity of 91% and 100% [61]. A subsequent study by Traube et al. confirmed its reliability in 111 patients, with a sensitivity of 94.1% and specificity of 79.2%, though specificity dropped to 51.2% in children with developmental delay. Inter-rater reliability varied by age, with κ = 0.60 overall and 0.85 in children aged two or older [62].

While developing CAP-D, researchers identified the need for targeted clinical training to improve the tool’s interpretive reliability and diagnostic accuracy by integrating pediatric and developmental expertise. To address this issue, a developmental anchor point chart spanning key ages (newborn, 4 weeks, 6 weeks, 8 weeks, 28 weeks, 1 year, and 2 years) was created and validated by a multidisciplinary panel, incorporating typical illness behaviors observed in hospitalized children. This chart was a key element in nurse training for the CAP-D validation trial and a practical bedside reference in clinical practice [63]. However, further studies with the CAP-D tool are needed in children less than or equal to 9 weeks old. In this population, the intraclass correlation coefficient (ICC) was 0.59, indicating poor to moderate reliability, and was notably lower compared to the ICC observed in infants older than 9 weeks, which was 0.72, reflecting moderate to good reliability. Among infants receiving MV, the ICC was 0.5, also representing poor to moderate reliability, and was significantly lower than the ICC of 0.69 found in those not on MV, which denoted moderate to good reliability. Fleiss’ kappa was 0.47 for all infants assessed, corresponding to slight to fair inter-rater agreement. Finally, applying anchor points did not enhance reliability [64].

One of the main advantages of the CAP-D lies in its practicality and speed. The CAP-D can be completed entirely through observational assessment. This makes it especially suitable for use in critically ill children, including those who are nonverbal, sedated, or developmentally delayed. Traube et al. highlight that the CAP-D takes less than two minutes to administer, facilitating routine screening during busy PICU workflows. Moreover, because it relies on nursing observations throughout the shift, it captures fluctuations in mental status more reliably than point-in-time assessments. Although its positive predictive value (PPV) is modest (around 54%), this is consistent with its intended use as a screening, not diagnostic, instrument. Its ease of use, minimal training requirements, and applicability across a wide pediatric age range have led several institutions to adopt CAP-D as their preferred screening tool for PD [62,65].

Assessments with the CAP-D can only be performed when the Richmond Agitation-Sedation Scale (RASS) score is between −3 and +4 or the State Behavioral Scale (SBS) is between −1 and +2 [62]. In screening for delirium in patients with developmental delay, if there was a RASS fluctuation of at least two points during a 24 h period along with a positive CAP-D, the specificity of diagnosis increased to 97%, the positive predictive value increased to 89%, and the negative predictive value remained similar at 87%, so this is superior than relying on the CAP-D alone [66]. The CAP-D is also available in multiple languages—Table 1.

### 6.4. Preschool and Pediatric Confusion Assessment Methods for the Intensive Care Unit

In 2011, Smith et al. introduced the Pediatric Confusion Assessment Method for the Intensive Care Unit (pCAM-ICU) to diagnose delirium in critically ill children aged five years and older without cognitive delays, whether mechanically ventilated or not. The original study included 68 patients with 146 assessments and showed high accuracy compared to DSM-IV-TR applied by psychiatrists, with 83% sensitivity, 99% specificity, and excellent inter-rater reliability (κ = 0.96) [7]. To address its limitations in younger children and those with developmental delays, Smith et al. later developed the Preschool Confusion Assessment Method for the Intensive Care Unit (psCAM-ICU), validated in 300 children aged 6 months to 5 years with 530 assessments, which showed 75% sensitivity, 91% specificity, and κ = 0.79 against DSM-IV-TR [30]. In 2021, the psCAM-ICU was also validated for infants under 6 months (49 patients and 189 assessments), with a sensitivity of 95%, specificity of 81%, and κ = 1.0 [82]. Both tools are objective, interactive adaptations of the adult Confusion Assessment Method for the Intensive Care Unit (CAM-ICU) and assess delirium using four core features: #1 acute/fluctuating mental status, #2 inattention, #3 altered level of consciousness, and #4 disorganized thinking. A diagnosis requires features #1 and #2, plus either #3 or #4. As with the CAP-D, assessments can only be performed when the RASS score is between −3 and +4 or the SBS is between −1 and +2 [7,30,83]. Finally, both take less than two minutes to be completed [8,22,34]. The advantages of psCAM-ICU and pCAM-ICU over CAP-D include their higher PPVs (78% and 88%, respectively, versus 54%) as diagnostic tools [7,30,65]. Versions in languages other than English are also available—Table 2.

### 6.5. Vanderbilt Assessment for Delirium in Infants and Children

Child psychiatrists and pediatric professionals developed the Vanderbilt Assessment for Delirium in Infants and Children (VADIC) to standardize delirium assessment by psychiatrists while validating the psCAM-ICU. The VADIC was developed because of the specific challenges of assessing delirium in hospitalized pediatric patients, including changes in baseline cognitive and language development; regression of previously learned skills during acute illness; inability of the patient to communicate verbally due to the presence of devices such as an enteral tube or endotracheal tube; differences in communication styles due to personal experience; and age-related separation anxiety. The VADIC uses key tasks to assess primary DSM criteria, such as attention, cognition, and arousal in pediatric patients of all ages. Furthermore, it organizes the clinical evaluation, encompassing the assessment of specific pediatric behaviors according to variations in development and cognitive variations, facilitating the diagnosis of delirium in hospitalized children. Members of the Special Interest Group (SIG) of the Committee on Physically Ill Children of the American Academy of Child and Adolescent Psychiatry (AACAP) verified the VADIC’s content validity, which represents a qualitative way to operationalize the psychiatric assessment, utilizing the DSM V as the current diagnostic standard [89].

### 6.6. Severity Scale for the Pediatric Confusion Assessment Method for the Intensive Care Unit

In the German validation study of the PAED and pCAM-ICU tools, Luetz et al. developed the Severity Scale for the Pediatric Confusion Assessment Method for the ICU (sspCAM-ICU), a tool adapted from the pCAM-ICU to quantify delirium severity. Scoring ranges from 0 (no delirium) to 19 (maximum severity), based on the intensity of inattention and altered mental status. In a cohort of 64 critically ill children aged five years or older, the sspCAM-ICU showed 85% sensitivity and 98% specificity against the DSM-IV-TR standard. In repeated assessments (*n* = 214), it maintained good diagnostic performance (sensitivity 71.8%, specificity 96.6%), outperforming the PAED scale significantly (*p* = 0.0008) [60].

### 6.7. Sophia Observation Withdrawal Symptoms Pediatric Delirium Scale

In 2017, Ista et al. published a preliminary validation study of the Sophia Observation Withdrawal Symptoms Pediatric Delirium scale (SOS-PD), adapted from the Sophia Observation Withdrawal Symptoms scale (SOS) initially developed for iatrogenic withdrawal assessment (IWS) in PICUs. SOS-PD assessments were conducted three times daily in a prospective observational study involving patients aged three months or older hospitalized for more than 48 h. A score ≥ 4 in two consecutive observations prompted evaluation by a child psychiatrist using DSM-IV criteria as the gold standard. The intraclass correlation coefficient for 16 observations was 0.90, and comparison of 63 paired assessments in 14 patients showed 96.8% sensitivity and 92% specificity. A follow-up study confirmed the tool’s inter-rater reliability, criterion validity, and construct validity (Pearson correlation with CAP-D: 0.89; *p* < 0.001), with an optimal cutoff of ≥4 points yielding a sensitivity of 92.3%, specificity of 96.5%, positive predictive value of 76.4%, and negative predictive value of 99.1%. SOS-PD assessments are not applicable when patients are in deep sedation or coma (RASS < −3 or Comfort-behavior score < 11) [90].

The SOS-PD is available in English, Dutch, Brazilian Portuguese, Czech, Danish, French, German, Italian, Japanese, Latin American Spanish, and Swedish and can be found via this link: https://www.comfortassessment.nl/web/index.php/instruments/sos-pediatric-delirium-sos-pd/ (accessed on 8 July 2025) [91].

### 6.8. Which Tools Are Recommended Today?

According to the 2022 Society of Critical Care Medicine Clinical Practice Guidelines on Prevention and Management of Pain, Agitation, Neuromuscular Blockade, and Delirium in Critically Ill Pediatric Patients With Consideration of the ICU Environment and Early Mobility (PANDEM Guidelines) of the Society of Critical Care Medicine (SCCM), the use of psCAM-ICU/pCAM-ICU or CAPD tools is recommended as they are the most valid and reliable delirium monitoring tools in critically ill pediatric patients (strength of recommendation: strong; quality of evidence: high) [33]. The authors also recommend routine screening for delirium in the PICU using a validated tool in critically ill pediatric patients at admission and until discharge or transfer from the ICU (strength of recommendation: strong; quality of evidence: high) at least twice daily [33]. The European Society of Paediatric and Neonatal Intensive Care (ESPNIC) also suggests them, highlighting the CAPD [34,40] with a grade of recommendation A and mentions the SOS-PD [40]. In addition, they recommend that delirium must be assessed and documented every 8–12 h (at least once per shift), 24–48 h after admission or as indicated by the delirium score of the clinical condition of the child, together with the vital signs [40]. Finally, a good practice statement of the Second Pediatric Acute Lung Injury Consensus Conference (PALICC-2) highlights that patients with pediatric acute respiratory distress syndrome (PARDS) should be assessed daily for delirium using a validated PD screening tool [92]—Table 3.

Despite the availability of bedside tools to screen/diagnose PD, numerous pediatric studies have recently shown that many of the ICUs globally have not adopted the national and international guidelines to diagnose, manage, and prevent delirium. Sperotto et al. found that, among 215 responding European PICUs, 58% systematically monitored delirium, mainly with the CAPD (48%) and SOS-PD (34%) scales, while 62% assessed iatrogenic withdrawal syndrome with the Withdrawal Assessment Tool-1 (WAT-1) (53%), with the most common treatments being dexmedetomidine or antipsychotics for delirium and rescue boluses for IWS; the adoption of analgesia-sedation protocols, present in 71% of PICUs, was associated with a higher probability of structured monitoring, guided weaning, and mobilization, indicating the importance of education and interprofessional work [93]. In Brazil, in an observational study conducted in three PICUs in Rio de Janeiro with 504 hospitalizations, Castro et al. showed that pediatric intensivists identified only two cases of delirium (0.4%), both with insufficient diagnostic criteria, suggesting that, in the absence of validated instruments, detection based solely on clinical experience leads to underdiagnosis or diagnostic error [94]. Another example is an international survey study conducted by Ista et al., which showed that only 44% of the 161 PICUs in North America, South America, and Europe routinely monitored for PD with a validated tool [95]. Finally, a study conducted in 28 United Kingdom and Ireland PICUs found that only 64% of the units implemented a PD screening protocol in 2023 despite large efforts for widespread PD education and training as well as building a national PD database [11]. These studies illustrate that, despite guidelines to routinely screen and diagnose PD to improve patient outcomes, buy-in from unit staff may be challenging, which reinforces the importance of frequent literature reviews and unit education among multidisciplinary staff.

## 7. Prevention and Treatment

The prevention and treatment of delirium have overlapping features. They go hand in hand because treatment can prevent subsequent episodes of delirium, and prevention techniques can mitigate the severity of delirium symptoms. Both approaches involve non-pharmacological care. However, the pharmacological approach is used only for treatment, not prevention [1,33,50]. Prevention of delirium in children admitted to NICU/PICU should be considered a clinical priority, especially given the high incidence and adverse outcomes associated with its occurrence. Among the most effective preventive strategies, minimizing exposure to deliriogenic drugs, such as benzodiazepines and anticholinergics, whose association with the development of delirium is already widely recognized in the literature, stands out. Reducing the use of these drugs, whenever possible, in addition to routinely reviewing the prescription with a focus on neuropsychiatric safety, constitutes a concrete and low-cost intervention that can significantly contribute to reducing the burden of delirium in critically ill pediatric patients [1,8,16,33].

### 7.1. Non-Pharmacological Approach

Non-pharmacological strategies for managing PD include educational, multicomponent, and technology-assisted interventions, often implemented as part of PICU bundles. Educational measures help children and caregivers overcome anxiety and maladaptive behaviors associated with PD. Multicomponent approaches integrate sleep–wake regulation, pain and agitation control, environmental adjustments, delirium screening, early mobilization, and parental involvement to enhance the prevention of delirium. Technology-assisted methods include maternal voice recordings, music therapy, and video distraction, aiming to reduce delirium, anxiety, sedative use, and length of stay [96]. In fact, regarding music therapy, the SCCM PANDEM Guidelines recommend that it can be offered to improve analgesia for critically ill postoperative pediatric patients (strong, moderate-level evidence). Music therapy has been shown to alleviate pain and anxiety, decrease the need for medications, lower inflammatory markers, and enhance both sleep quality and physical mobility, common risk factors for delirium in the PICU [33].

Maintaining a consistent daily routine, adjusting light exposure, promoting adequate sleep, and encouraging family presence are key elements [97]. Other interventions include the use of eyeglasses and hearing aids, minimizing noise, cognitive orientation, and facilitating physical activity. Bundles such as the Bundle to Eliminate Delirium (BED) have demonstrated reduced noise pollution in the PICU, which could theoretically improve sleep quality and help mitigate risk factors for delirium [38,39,41]. Sleep quality remains a major concern, with ongoing efforts to improve monitoring and interventions [98].

Implementation studies of early rehabilitation bundles have demonstrated reductions in sedative use and enhanced mobilization without compromising safety [99]. However, current evidence is insufficient to identify the most effective non-pharmacological interventions for PD [96]. The PANDEM Guidelines recommend facilitating family presence, using noise-reducing devices, promoting sleep hygiene, and early mobilization based on conditional recommendations supported by low-quality evidence [33].

Additionally, the A-F Bundle from the SCCM ICU Liberation initiative (covering pain management, spontaneous awakening and breathing trials, choice of analgesia and sedation, delirium management, early mobility, and family engagement) has been associated with lower delirium incidence and shorter ICU stays in adults [100]. Quality improvement projects have shown that structured bundles can significantly reduce delirium rates and improve patient outcomes in the PICU setting [101,102].

### 7.2. Pharmacological Approach

In critically ill pediatric patients, minimizing benzodiazepine use is strongly recommended to reduce the incidence, duration, or severity of delirium. Additionally, reducing overall sedation exposure is suggested to lessen the risk of coma and delirium, although the supporting evidence is limited. This can be achieved by establishing an individualized RASS or SBS target for each patient, reviewing the target daily, and titrating sedative infusions accordingly to ensure that patients are not oversedated [37]. Moreover, as mentioned previously, medications such as anticholinergics, antiemetics, antiepileptics, corticosteroids, opioids, dexmedetomidine, inotropes, and vasoactive drugs are likely deliriogenic [26,36,40,42].

Regarding dexmedetomidine, the most recent update of the SCCM guidelines for critically ill adults recommends its use over propofol for sedation in mechanically ventilated ICU patients when light sedation or delirium reduction is prioritized [103]. Additionally, studies suggest that dexmedetomidine may prevent or shorten the duration of delirium in adults, although its therapeutic role in established delirium remains uncertain [104]. A recent target trial emulation in ICU patients showed that early dexmedetomidine use was associated with greater agitation resolution, higher extubation rates, and reduced tracheostomy, with consistent effects across subgroups including non-ventilated patients, supporting its use for psychomotor agitation, but not for hypoactive delirium, where no benefit has been demonstrated [105]. For pediatric patients, the PANDEM guidelines advise considering dexmedetomidine as a primary sedative in postoperative cardiac surgical children expected to undergo early extubation (strong recommendation; moderate evidence) and to reduce the risk of tachyarrhythmias (conditional recommendation; low evidence) [33]. Dexmedetomidine significantly reduces pediatric emergence agitation and delirium after anesthesia, findings that parallel adult data in ICU hyperactive delirium [106].

Although frequently prescribed in clinical practice [107], currently, no recommendation can be made for or against the use of antipsychotics compared to usual care for the treatment or prevention of delirium in adults [103] or children in the ICU [33]. Antipsychotics consist of a symptomatic medication for agitation, and the most recent guidelines suggest that antipsychotics should be used only after predisposing and precipitating risk factors have been addressed and non-pharmacological measures have been used. If use is strictly necessary, the antipsychotic should be administered in low doses for the shortest possible time, with monitoring for side effects [33,103,107,108].

Antipsychotic agents are categorized as either typical (first-generation) or atypical (second-generation) according to their mechanisms of action and associated side effect profiles. Among atypical antipsychotics, distinctions in receptor binding affinities lead to differential neurotransmitter modulation and adverse effect patterns, which may influence their effectiveness in managing delirium [109]. Haloperidol is a typical antipsychotic that has been used clinically for many years, is the most widely described in the literature, and can be administered orally (PO) or intravenously (IV). Intramuscular (IM) administration is generally avoided in pediatric patients. Since IV haloperidol avoids first passage through the liver, it likely causes less dystonia, but the risk of arrhythmia remains high [110]. Haloperidol may improve agitation in children but may also put patients at risk of developing several adverse effects, even at low plasma concentrations and at recommended doses [111]. Risperidone, olanzapine, and quetiapine are the most studied atypical antipsychotics and are currently the first choice for the control of PD. Risperidone is equipotent to haloperidol and is available in tablets and orally disintegrating solutions that make it useful in infants. Olanzapine and quetiapine have been described in the treatment of delirium in critically ill infants, children, and adolescents. Quetiapine appears to be less associated with hepatic complications, useful in patients with hepatic impairment, before or after liver transplantation [110,112]—Table 4.

According to the PANDEM Guidelines, the routine use of haloperidol or atypical antipsychotics for delirium prevention or reduction is not recommended. However, in cases of refractory delirium with severe symptoms, these medications may be considered, with careful attention to potential adverse effects. For patients receiving such agents, a baseline ECG and ongoing monitoring of electrolytes and QTc interval are strongly advised [30]. In pediatrics, the use of quetiapine has been reported safely in delirium management, with a dosage of 0.5 mg/kg/dose every eight hours [114,118,119,120].

In summary, although these pharmacologic agents are used in clinical practice for the management of PD, including dexmedetomidine and antipsychotics, there is currently no established consensus or clear evidence-based guideline supporting their routine use in the neonatal and pediatric populations. The decision to initiate pharmacologic treatment should always follow a careful assessment of risk factors and should prioritize non-pharmacologic strategies as first-line interventions [33,121]. Studies show that polypharmacy is an independent risk factor for delirium, and therefore, a daily review of each prescription with consequent reduction in medications has been suggested [108,109]. Therefore, prevention remains the cornerstone of PD management, and minimizing exposure to deliriogenic medications is a key preventive strategy. Careful daily review of all prescribed drugs, with a focus on reducing the use of benzodiazepines, anticholinergics, corticosteroids, and other agents known to contribute to delirium risk, is strongly recommended. Implementing individualized sedation targets, avoiding polypharmacy, and optimizing pain and agitation control through non-pharmacologic interventions can significantly reduce delirium incidence. A proactive, multidisciplinary approach to medication stewardship is essential to creating a NICU/PICU that supports cognitive recovery and reduces the likelihood of delirium onset [1,33,121].

## 8. Conclusions

Despite historic acknowledgment of the existence of delirium in children, it has only recently been recognized as relevant in pediatrics. A more standardized assessment worldwide has been possible thanks to the development of reliable and valid diagnostic tools and translation processes, as well as the cross-cultural adaptation, validity, and reliability of these instruments in other languages. However, there are still many obstacles to be overcome, especially in dispelling the idea that delirium is just a behavior, but rather that it is a syndrome of acute brain dysfunction that deserves targeted evaluation and immediate intervention.

## Figures and Tables

**Table 1:** Psychometric properties of CAP-D in non-English versions.

Language/Country	Population (*n*)	Sensitivity (%)	Specificity (%)	Cronbach’s α	CRI	ICC	Inter-Rater Reliability (k)
Chinese/China	250 patients	96.7	93.1	0.819	-	0.835	-
Danish/Denmark	30 patients/92 assessments	-	-	-	-	-	0.85
French/France	25 patients	-	-	-	-	-	0.92
Greek	35 patients					0.97	
Italian/Italy [1]	42 patients	-	-	0.96	0.94	-	-
Italian/Italy [2]	70 patients	93	56	-	-	Intra-rater 0.98 Inter-rater 0.93	-
Japanese/Japan	41 patients/92 assessments Mechanical ventilation	90 97	88 64	-	-	-	0.89
Korean/Korea	50 patients	93.8	75	0.91		0.98	
Swedish/Sweden	10 Registered nurses	-	-	-	-	0.857	-
Turkish/Turkey	76 patients	100	95	-	-	-	Nurses: 0.74 Psychiatrists: 0.86

Legend: CRI—Composite Reliability Index; ICC—Intraclass Correlation Coefficient. Note: the following versions were translated and/or cross-culturally adapted but have not yet been validated: Brazilian Portuguese, German, Persian, Spanish. Sources: [67,68,69,70,71,72,73,74,75,76,77,78,79,80,81].

**Table 2:** Psychometric properties of the available non-English versions of the psCAM-ICU and pCAM-ICU tools.

Tool	Language/Country	Population (*n*)	Sensitivity (%)	Specificity (%)	Inter-Rater Reliability (k)
pCAM-ICU	German/Germany	64 patients/214 assessments Repeated assessments	76.9 52.3	98	-
	Portuguese/Brazil	116 patients/149 assessments	90.9	99.3	1.0
psCAM-ICU	Japanese/Japan	19 patients/56 assessments	90	93	0.92
	Spanish/Colombia	31 patients/31 assessments	93.3	94.8	0.78

Notes: The Japanese and the Spanish (Colombia) versions of pCAM-ICU have been translated and cross-culturally adapted, but have not yet been validated. The psCAM-ICU was first translated into Spanish by researchers from Puerto Rico as psCAM-ICU-S. Sources: [8,60,84,85,86,87,88].

**Table 3:** Validated and recommended tools for delirium screening in the PICU.

	psCAM-ICU	pCAM-ICU	CAP-D	SOS-PD
Age	Neonates to 5 yr	≥5 yr	Neonates to 21 yr	0 to 18 yr
Patients with cognitive developmental delay	No	No	Yes	Included patients with a mild or transient history of developmental problems
Patients on MV	Yes	Yes	Yes	Yes
Cut-off points	No score ranges Presence of features 1 and 2 and 3 or 4	No score ranges Presence of features 1 and 2 and 3 or 4	≥9	≥4
Sensitivity (%)	Neonates to 6 months: 95 6 months to 5 yr: 91	83	94.1	92.3
Specificity (%)	Neonates to 6 months: 81 6 months to 5 yr: 75	99	79.2	96.5
Inter-rater reliability (κ)	Neonates to 6 months: 1.0 6 months to 5 yr: 0.79	0.96	0.94	0.79 to 1.0

Legend: yr—year; MV—mechanical ventilation; CAP-D—Cornell Assessment of Pediatric Delirium; pCAM-ICU—Pediatric Confusion Assessment Method for the Intensive Care Unit; psCAM-ICU—PreSchool Confusion Assessment Method for the Intensive Care Unit; SOS-PD—Sophia Observation Withdrawal Symptoms—Pediatric Delirium Scale. Note: the validation studies were based on the gold-standard analysis using the Diagnostic and Statistical Manual of Mental Disorders, fourth edition, of the American Psychiatry Association (APA), except for psCAM-ICU, in which the fifth edition (DSM-5) was used. Sources: [7,30,62,90].

**Table 4:** Common antipsychotics to manage delirium in children.

Antipsychotic	Formulation	Dose	Observations
Haloperidol	Oral liquid and oral tablet, IV, IM	0.05 mg/kg/day divided twice daily	It is the only antipsychotic that has a parenteral formulation, which is an advantage in patients who cannot use the enteral route. IV administration is approximately twice as potent as PO.
Risperidone	Oral liquid, oral tablet, disintegrating tablet IM	<5 yr: 0.1 mg once or twice daily ≥5 yr: 0.2 mg once or twice daily	It is the only liquid formulation, facilitating oral administration. Sedation is common. Risk of orthostatic hypotension. For the management of PD, IM administration has not yet been explored.
Olanzapine	Oral tablet, IM	Infants: 0.625 mg per oral qHS to twice daily Toddlers: 1.25 mg per oral qHS to twice daily Older, larger, or extremely agitated patient: scheduled starting dose of 2.5 mg to 5 mg per oral qHS to twice daily	Its administration was associated with the elevation of hepatic transaminases. For the management of PD, IM administration has not yet been explored.
Quetiapine	Oral tablet	0.5 mg/kg/dose every eight hours	It has also been used in the NICU. Risk of orthostatic hypotension.

Legend: IM—intramuscular; IV—intravenous; NICU—Neonatal Intensive Care Unit; PD—pediatric delirium; PO—oral route; qHS—every night at bedtime. Note: all antipsychotics are prescribed off-label. Sources: [113,114,115,116,117].

## Data Availability

Not applicable.

## Abbreviations

IM—intramuscular; IV—intravenous; NICU—Neonatal Intensive Care Unit; PD—pediatric delirium; PO—oral route; qHS—every night at bedtime; yr—year; MV—mechanical ventilation; CAP-D—Cornell Assessment of Pediatric Delirium; pCAM-ICU—Pediatric Confusion Assessment Method for the Intensive Care Unit; psCAM-ICU—PreSchool Confusion Assessment Method for the Intensive Care Unit; SOS-PD—Sophia Observation Withdrawal Symptoms—Pediatric Delirium Scale; CRI—Composite Reliability Index; ICC—Intraclass Correlation Coefficient.

## References

[1] Page V., Ely E.W. (2015). Delirium in Critical Care.

[2] Smith H.A.B., Williams S.R., Hughes C.G., Pandharipande P.P., Ely E.W. (2020). Pediatric delirium assessment, prevention, and management. Delirium: Acute Brain Dysfunction in the Critically Ill.

[3] Adamis D., Treloar A., Martin F.C., Macdonald A.J. (2007). A brief review of the history of delirium as a mental disorder. Hist. Psychiatry.

[4] Bleuler E. (1923). Textbook of Psychiatry.

[5] Schieveld J.N.M., Ista E., Knoester H., Molag M.L., Rey J.M. (2015). Delirium. IACAPAP e-Textbook of Child and Adolescent Mental Health.

[6] Kanner L. (1935). Child Psychiatry.

[7] Smith H.A., Boyd J., Fuchs D.C., Melvin K., Berry P., Shintani A., Eden S.K., Terrell M.K., Boswell T., Wolfram K. (2011). Diagnosing delirium in critically ill children: Validity and reliability of the Pediatric Confusion Assessment Method for the Intensive Care Unit. Crit. Care Med..

[8] Traube C., Mauer E.A., Gerber L.M., Kaur S., Joyce C., Kerson A., Carlo C., Notterman D., Worgall S., Silver G. (2016). Cost Associated with Pediatric Delirium in the ICU. Crit. Care Med..

[9] Lei L., Li Y., Xu H., Zhang Q., Wu J., Zhao S., Zhang X., Xu M., Zhang S. (2023). Incidence, associated factors, and outcomes of delirium in critically ill children in China: A prospective cohort study. BMC Psychiatry.

[10] AlDaithan A., Shaheen N., Alahmari E., Smari A.A., Al Ahmadi A., Almalahi A., Alotaibi M., AlGhuraibi A., Alhusaini A., Bin Shaman A. (2024). Age-specific vulnerability and high prevalence of delirium in pediatric intensive care based on a prospective cohort study. Sci. Rep..

[11] Blackwood B., Aitken L.M., Craske J., Gala-Peralta S., Liew A., Murray M., McIlmurray L., Tume L.N. (2025). National screening for delirium in paediatric intensive care units: A quality improvement initiative. Nurs. Crit. Care.

[12] Dervan L.A., Killien E.Y., Smith M.B., Watson R.S. (2022). Health-Related Quality of Life Following Delirium in the PICU. Pediatr. Crit. Care Med..

[13] Manning J.C., Pinto N.P., Rennick J.E., Colville G., Curley M.A.Q. (2018). Conceptualizing Post Intensive Care Syndrome in Children-The PICS-p Framework. Pediatr. Crit. Care Med..

[14] Lim J.K.B., Marimuttu V.J., Lee J.H. (2023). Delirium: The Next Vital Sign in the PICU?. Pediatr. Crit. Care Med..

[15] Davis D.H., Kreisel S.H., Muniz Terrera G., Hall A.J., Morandi A., Boustani M., Neufeld K.J., Lee H.B., Maclullich A.M., Brayne C. (2013). The epidemiology of delirium: Challenges and opportunities for population studies. Am. J. Geriatr. Psychiatry.

[16] Wilson J.E., Mart M.F., Cunningham C., Shehabi Y., Girard T.D., MacLullich A.M.J., Slooter A.J.C., Ely E.W. (2020). Delirium. Nat. Rev. Dis. Primers.

[17] Meyburg J., Dill M.L., von Haken R., Picardi S., Westhoff J.H., Silver G., Traube C. (2018). Risk Factors for the Development of Postoperative Delirium in Pediatric Intensive Care Patients. Pediatr. Crit. Care Med..

[18] Castro R.E.V., Prata-Barbosa A., de Magalhães-Barbosa M.C., Cunha A.J.L.A.D., Cheniaux E., Smith H.A.B. (2020). Validity and Reliability of the Brazilian Portuguese Version of the Pediatric Confusion Assessment Method for the ICU. Pediatr. Crit. Care Med..

[19] Semple D., Howlett M.M., Strawbridge J.D., Breatnach C.V., Hayden J.C. (2022). A Systematic Review and Pooled Prevalence of Delirium in Critically Ill Children. Crit. Care Med..

[20] Traube C., Silver G., Reeder R.W., Doyle H., Hegel E., Wolfe H.A., Schneller C., Chung M.G., Dervan L.A., DiGennaro J.L. (2017). Delirium in Critically Ill Children: An International Point Prevalence Study. Crit. Care Med..

[21] Staveski S.L., Pickler R.H., Khoury P.R., Ollberding N.J., Donnellan A.L., Mauney J.A., Lincoln P.A., Baird J.D., Gilliland F.L., Merritt A.D. (2021). Prevalence of ICU Delirium in Postoperative Pediatric Cardiac Surgery Patients. Pediatr. Crit. Care Med..

[22] Siegel E.J., Groves A.M., Silver G., Hojsak J., Lim C.A., Traube C. (2021). Delirium in the NICU: A Point Prevalence Study. Hosp. Pediatr..

[23] Flores A.E.R., Oura K.H.U., Rocha P.K., Belela-Anacleto A.S.C., Kusahara D.M. (2021). Incidence and Factors Associated with Delirium in Children in a Single Pediatric Intensive Care Unit in Brazil. J. Pediatr. Nurs..

[24] Difabio B., McGetrick M., Campbell C., Kelly B., Munoz-Pareja J. (2019). Use of the Cornell Assessment of Pediatric Delirium (CAPD) for identifying delirium and associated risk factors in the pediatric intensive care unit. Pediatrics.

[25] Patel A.K., Biagas K.V., Clark E.C., Traube C. (2017). Delirium in the Pediatric Cardiac Extracorporeal Membrane Oxygenation Patient Population: A Case Series. Pediatr. Crit. Care Med..

[26] Gray M.C., Traube C., Sewell T.B., Geneslaw A.S. (2024). Delirium Associated with COVID-19 in Critically ill Children: An Observational Cohort Study. J. Intensive Care Med..

[27] Potter K.M., Kennedy J.N., Onyemekwu C., Prendergast N.T., Pandharipande P.P., Ely E.W., Seymour C., Girard T.D. (2024). Data-derived subtypes of delirium during critical illness. EBioMedicine.

[28] Pinzón-Casas E.Y., Henao-Castaño Á.M., Fajardo-Ramos E. (2020). Characteristics of delirium in the pediatric intensive care unit using the dynamic symptoms model. Salud Barranquilla.

[29] la Cour K.N., Andersen-Ranberg N.C., Weihe S., Poulsen L.M., Mortensen C.B., Kjer C.K.W., Collet M.O., Estrup S., Mathiesen O. (2022). Distribution of delirium motor subtypes in the intensive care unit: A systematic scoping review. Crit. Care.

[30] Smith H.A., Gangopadhyay M., Goben C.M., Jacobowski N.L., Chestnut M.H., Savage S., Rutherford M.T., Denton D., Thompson J.L., Chandrasekhar R. (2016). The Preschool Confusion Assessment Method for the ICU: Valid and Reliable Delirium Monitoring for Critically Ill Infants and Children. Crit. Care Med..

[31] Schieveld J.N., Lousberg R., Berghmans E., Smeets I., Leroy P.L., Vos G.D., Nicolai J., Leentjens A.F., van Os J. (2008). Pediatric illness severity measures predict delirium in a pediatric intensive care unit. Crit. Care Med..

[32] Patel A.K., Bell M.J., Traube C. (2017). Delirium in Pediatric Critical Care. Pediatr. Clin. N. Am..

[33] Smith H.A.B., Besunder J.B., Betters K.A., Johnson P.N., Srinivasan V., Stormorken A., Farrington E., Golianu B., Godshall A.J., Acinelli L. (2022). 2022 Society of Critical Care Medicine Clinical Practice Guidelines on Prevention and Management of Pain, Agitation, Neuromuscular Blockade, and Delirium in Critically Ill Pediatric Patients with Consideration of the ICU Environment and Early Mobility. Pediatr. Crit. Care Med..

[34] Ovchinsky N., Frazier W., Auletta J.J., Dvorak C.C., Ardura M., Song E., McArthur J., Jeyapalan A., Tamburro R., Mahadeo K.M. (2018). Consensus Report by the Pediatric Acute Lung Injury and Sepsis Investigators and Pediatric Blood and Marrow Transplantation Consortium Joint Working Committees on Supportive Care Guidelines for Management of Veno-Occlusive Disease in Children and Adolescents, Part 3: Focus on Cardiorespiratory Dysfunction, Infections, Liver Dysfunction, and Delirium. Biol. Blood Marrow Transplant..

[35] Nellis M.E., Goel R., Feinstein S., Shahbaz S., Kaur S., Traube C. (2018). Association Between Transfusion of RBCs and Subsequent Development of Delirium in Critically Ill Children. Pediatr. Crit. Care Med..

[36] Ista E., Traube C., de Neef M., Schieveld J., Knoester H., Molag M., Kudchadkar S.R., Strik J., Dutch Multidisciplinary Pediatric Delirium Guideline Group (2023). Factors Associated with Delirium in Children: A Systematic Review and Meta-Analysis. Pediatr. Crit. Care Med..

[37] Madden K., Hussain K., Tasker R.C. (2018). Anticholinergic Medication Burden in Pediatric Prolonged Critical Illness: A Potentially Modifiable Risk Factor for Delirium. Pediatr. Crit. Care Med..

[38] Kawai Y., Weatherhead J.R., Traube C., Owens T.A., Shaw B.E., Fraser E.J., Scott A.M., Wojczynski M.R., Slaman K.L., Cassidy P.M. (2019). Quality Improvement Initiative to Reduce Pediatric Intensive Care Unit Noise Pollution with the Use of a Pediatric Delirium Bundle. J. Intensive Care Med..

[39] Kalvas L.B., Harrison T.M., Curley M.A.Q., Ordway M.R., Redeker N.S., Happ M.B. (2023). An observational pilot study of sleep disruption and delirium in critically ill children. Heart Lung.

[40] Harris J., Ramelet A.S., van Dijk M., Pokorna P., Wielenga J., Tume L., Tibboel D., Ista E. (2016). Clinical recommendations for pain, sedation, withdrawal and delirium assessment in critically ill infants and children: An ESPNIC position statement for healthcare professionals. Intensive Care Med..

[41] Weatherhead J.R., Niedner M., Dahmer M.K., Malas N., Owens T., Kawai Y. (2022). Patterns of Delirium in a Pediatric Intensive Care Unit and Associations with Noise Pollution. J. Intensive Care Med..

[42] Alasad J.A., Abu Tabar N., Ahmad M.M. (2015). Patients’ experience of being in intensive care units. J. Crit. Care..

[43] Diseroad E.R., Minnick S., Hutson T.K. (2024). Assessment of intensive care unit delirium in developmentally delayed children. Pediatr. Res..

[44] McAlinden B., Pool N., Harnischfeger J., Waak M., Campbell M. (2024). ‘Baby Liberation’-Developing and implementing an individualised, developmentally-supportive care bundle to critically unwell infants in an Australian Paediatric Intensive Care Unit. Early Hum. Dev..

[45] Smith M.B., Killien E.Y., Watson R.S., Dervan L.A. (2025). Family Presence at the PICU Bedside and Pediatric Patient Delirium: Retrospective Analysis of a Single-Center Cohort, 2014–2017. Pediatr. Crit. Care Med..

[46] Maldonado J.R. (2018). Delirium pathophysiology: An updated hypothesis of the etiology of acute brain failure. Int. J. Geriatr. Psychiatry..

[47] American Psychiatric Association (2022). Diagnostic and Statistical Manual of Mental Disorders.

[48] Turkel S.B., Tavaré C.J. (2003). Delirium in children and adolescents. J. Neuropsychiatry Clin. Neurosci..

[49] Schieveld J.N., van der Valk J.A., Smeets I., Berghmans E., Wassenberg R., Leroy P.L., Vos G.D., van Os J. (2009). Diagnostic considerations regarding pediatric delirium: A review and a proposal for an algorithm for pediatric intensive care units. Intensive Care Med..

[50] Bettencourt A., Mullen J.E. (2017). Delirium in Children: Identification, Prevention, and Management. Crit. Care Nurse.

[51] Mosharaf M.P., Alam K., Gow J., Mahumud R.A. (2025). Cytokines and inflammatory biomarkers and their association with post-operative delirium: A meta-analysis and systematic review. Sci. Rep..

[52] Levey A.S., Eckardt K.U., Dorman N.M., Christiansen S.L., Hoorn E.J., Ingelfinger J.R., Inker L.A., Levin A., Mehrotra R., Palevsky P.M. (2020). Nomenclature for kidney function and disease: Report of a Kidney Disease: Improving Global Outcomes (KDIGO) Consensus Conference. Kidney Int..

[53] Turkel S.B., Braslow K., Tavaré C.J., Trzepacz P.T. (2003). The delirium rating scale in children and adolescents. Psychosomatics.

[54] Calandriello A., Tylka J.C., Patwari P.P. (2018). Sleep and Delirium in Pediatric Critical Illness: What Is the Relationship?. Med. Sci..

[55] Trzepacz P.T., Mittal D., Torres R., Kanary K., Norton J., Jimerson N. (2001). Validation of the Delirium Rating Scale-revised-98: Comparison with the delirium rating scale and the cognitive test for delirium. J. Neuropsychiatry Clin. Neurosci..

[56] Boettger S., Nuñez D.G., Meyer R., Richter A., Fernandez S.F., Rudiger A., Schubert M., Jenewein J. (2017). Delirium in the intensive care setting and the Richmond Agitation and Sedation Scale (RASS): Drowsiness increases the risk and is subthreshold for delirium. J. Psychosom. Res..

[57] Janssen N.J., Tan E.Y., Staal M., Janssen E.P., Leroy P.L., Lousberg R., van Os J., Schieveld J.N. (2011). On the utility of diagnostic instruments for pediatric delirium in critical illness: An evaluation of the Pediatric Anesthesia Emergence Delirium Scale, the Delirium Rating Scale 88, and the Delirium Rating Scale-Revised R-98. Intensive Care Med..

[58] Sikich N., Lerman J. (2004). Development and psychometric evaluation of the pediatric anesthesia emergence delirium scale. Anesthesiology.

[59] Blankespoor R.J., Janssen N.J., Wolters A.M., Van Os J., Schieveld J.N. (2012). Post-hoc revision of the pediatric anesthesia emergence delirium rating scale: Clinical improvement of a bedside-tool?. Minerva Anestesiol..

[60] Luetz A., Gensel D., Müller J., Weiss B., Martiny V., Heinz A., Wernecke K.D., Spies C. (2016). Validity of Different Delirium Assessment Tools for Critically Ill Children: Covariates Matter. Crit. Care Med..

[61] Silver G., Traube C., Kearney J., Kelly D., Yoon M.J., Nash Moyal W., Gangopadhyay M., Shao H., Ward M.J. (2012). Detecting pediatric delirium: Development of a rapid observational assessment tool. Intensive Care Med..

[62] Traube C., Silver G., Kearney J., Patel A., Atkinson T.M., Yoon M.J., Halpert S., Augenstein J., Sickles L.E., Li C. (2014). Cornell Assessment of Pediatric Delirium: A valid, rapid, observational tool for screening delirium in the PICU. Crit. Care Med..

[63] Silver G., Kearney J., Traube C., Hertzig M. (2015). Delirium screening anchored in child development: The Cornell Assessment for Pediatric Delirium. Palliat. Support. Care.

[64] Cleveland M., Baute R., Clindaniel C., Hertz L., Pond R., Centers G.I. (2023). Inter-Rater Reliability of Delirium Screening of Infants in the Cardiac ICU: A Prospective, Observational Study. Pediatr. Crit. Care Med..

[65] Paterson R.S., Kenardy J.A., Dow B.L., De Young A.C., Pearson K., Aitken L.M., Long D.A. (2021). Accuracy of delirium assessments in critically ill children: A prospective, observational study during routine care. Aust. Crit. Care.

[66] Kaur S., Silver G., Samuels S., Rosen A.H., Weiss M., Mauer E.A., Geber L.M., Greenwald B.M., Traube C. (2020). Delirium and Developmental Disability: Improving Specificity of a Pediatric Delirium Screen. Pediatr. Crit. Care Med..

[67] Barbosa M.D.S.R., Duarte M.D.C.M.B., Bastos V.C.S., Andrade L.B. (2018). Translation and cross-cultural adaptation of the Cornell Assessment of Pediatric Delirium scale for the Portuguese language. Rev. Bras. Ter. Intensiva..

[68] Barbosa M.D.S.R., Andrade L.B., Duarte M.D.C.M.B., Castro R.E.V. (2023). Translation and cross-cultural adaptation of the anchor points of the Cornell Assessment of Pediatric Delirium scale into Portuguese. Crit. Care Sci..

[69] Dill M.L., von Haken R., Traube C., Silver G., Reyna M., Asaro L.A., Worgall S. (2016). Erfassung eines Delirs bei pädiatrischen Intensivpatienten. Monatsschr. Kinderheilkd..

[70] Navaeifar M.R., Abbaskhanian A., Shahbaznejad L., Khoshkam M. (2019). Translation, adaptation and validity assessment of the Cornell Assessment of Pediatric Delirium Scale in Persian language. J. Mazandaran Univ. Med. Sci..

[71] Fernández-Carrión F., González-Salas E., Silver G., Traube C. (2019). Translation and Cultural Adaptation of Cornell Assessment of Pediatric Delirium to Spanish. Pediatr. Crit. Care Med..

[72] He S., Wang Y.L., Zuo Z.L. (2019). Clinical application of the Chinese version of Cornell assessment of pediatric delirium: A pilot study. Zhonghua Er Ke Za Zhi.

[73] Simonsen B.Y., Lisby M., Traube C., Skovby P. (2019). The Cornell Assessment of Pediatric Delirium: Translation and inter-rater reliability in a Danish pediatric intensive care unit. Acta Anaesthesiol. Scand..

[74] De Cloedt L., Harrington K., Du Pont-Thibodeau G., Ducharme-Crevier L. (2019). Translation and validation of a French language version of the Cornell Assessment of Pediatric Delirium tool. Méd. Intensive Réa..

[75] Hoshino H., Matsuishi Y., Enomoto Y., Shimojo N., Kido T., Matsuzaki A., Matsubara M., Kato H., Hoshino T., Traube C. (2020). The Validity and Reliability of the Japanese Version of the Cornell Assessment of Pediatric Delirium. Pediatr. Crit. Care Med..

[76] Simeone S., Rea T., Gargiulo G., Esposito M.R., Guillari A., Traube C., Silver G.H., Pucciarelli G. (2019). Cornell Assessment of Pediatric Delirium: Italian cultural validation and preliminary testing. Prof. Inferm..

[77] Fazio P.C., Daverio M., Masola M., D’Angelo I., Frison S., Zaggia C., Simeone S., Pucciarelli G., Gregori D., Comoretto R. (2022). Italian Version of the Cornell Assessment of Pediatric Delirium: Evaluation of the Scale Reliability and Ability to Detect Delirium Compared to Pediatric Intensive Care Unit Physicians Clinical Evaluation. Front. Pediatr..

[78] Jang H.J., Kim J.H. (2020). Validation of the Korean version of the Cornell Assessment of Pediatric Delirium. J. Korean Clin. Nurs. Res..

[79] Åkerman S., Axelin A., Traube C., Frithiof R., Thernström Blomqvist Y. (2024). Adapting the Cornell assessment of pediatric delirium for Swedish context: Translation, cultural validation and inter-rater reliability. BMC Pediatr..

[80] Volanaki A., Briassoulis G., Gerostergios G., Samiotakis G., Soumaki E., Traube C., Ilia S. (2024). Adaptation and Validation of the Cornell Assessment of Pediatric Delirium Tool in the Greek Language. Pediatr. Crit. Care Med..

[81] Uyar E., Kutlu N.O., Akçay E., Dinç G., Onat M., Koçkuzu E., Meral Y., Traube C. (2025). Cornell Assessment of Pediatric Delirium: Turkish translation and validation. Turk. J. Pediatr..

[82] Canter M.O., Tanguturi Y.C., Ellen Wilson J., Williams S.R., Exum S.A., Umrania H.M., Betters K.A., Raman R., Ely E.W., Pandharipande P.P. (2021). Prospective Validation of the Preschool Confusion Assessment Method for the ICU to Screen for Delirium in Infants Less Than 6 Months Old. Crit. Care Med..

[83] ICU Delirium & Cognitive Impairment Study Group (2025). Pediatric Care [Internet].

[84] Tsuruta R., Yamase H. (2011). Investigation of delirium in critically ill children: Japanese version of the Pediatric Confusion Assessment Method for the Intensive Care Unit (pCAM-ICU). Jpn. Assoc. Crit. Care Nurs..

[85] Franco J.G., Ricardo C., Muñoz J.F., de Pablo J., Berry P., Ely E.W., Smith H.A. (2012). Diagnosing delirium in critically ill children: Spanish translation and cultural adaptation of the Pediatric Confusion Assessment Method for the Intensive Care Unit. Crit. Care Med..

[86] Figueroa-Ramos M.I., Arroyo-Novoa C.M., García-DeJesús R.L., Sepúlveda-Santiago C.S., Solís-Báez S.S., Ely E.W., Smith H. (2020). Translation and cultural adaptation process to Spanish of the Preschool Confusion Assessment Method for the Intensive Care Unit. Med. Intensiva. (Engl. Ed.).

[87] Pinzón-Casas E., Soto-Trujillo M., Camargo-Agón L., Henao-Castaño Á., Gualdrón N., Bonilla-González C. (2021). Preschool Confusion Assessment Method for the Intensive Care Unit-Spanish (psCAM-ICU-S): Cross-Cultural Adaptation and Validation in Colombia. Front. Pediatr..

[88] Matsuishi Y., Hoshino H., Shimojo N., Enomoto Y., Kido T., Matsuzaki A., Mathis B.J., Kawano S., Inoue Y. (2019). Verifying the Japanese version of the Preschool Confusion Assessment Method for the ICU (psCAM-ICU). Acute Med. Surg..

[89] Gangopadhyay M., Smith H., Pao M., Silver G., Deepmala D., De Souza C., Garcia G., Giles L., Denton D., Jacobowski N. (2017). Development of the Vanderbilt Assessment for Delirium in Infants and Children to Standardize Pediatric Delirium Assessment by Psychiatrists. Psychosomatics.

[90] Ista E., van Beusekom B., van Rosmalen J., Kneyber M.C.J., Lemson J., Brouwers A., Dieleman G.C., Dierckx B., de Hoog M., Tibboel D. (2018). Validation of the SOS-PD scale for assessment of pediatric delirium: A multicenter study. Crit. Care.

[91] Erasmus MC–Sophia Children’s Hospital (2025). SOS-Pediatric Delirium (SOS-PD) Scale [Internet].

[92] Valentine S.L., Kudchadkar S.R., Ward S., Morrow B.M., Nadkarni V.M., Curley M.A.Q., Second Pediatric Acute Lung Injury Consensus Conference (PALICC-2) of the Pediatric Acute Lung Injury and Sepsis Investigators (PALISI) Network (2023). Nonpulmonary Treatments for Pediatric Acute Respiratory Distress Syndrome: From the Second Pediatric Acute Lung Injury Consensus Conference. Pediatr. Crit. Care Med..

[93] Sperotto F., Ramelet A.S., Daverio M., Mondardini M.C., von Borell F., Brenner S., Tibboel D., Ista E., Pokorna P., Amigoni A. (2023). Assessment and management of iatrogenic withdrawal syndrome and delirium in pediatric intensive care units across Europe: An ESPNIC survey. Pharmacotherapy.

[94] de Castro R.E.V., de Magalhães-Barbosa M.C., Cunha A.J.L.A.D., Cheniaux E., Prata-Barbosa A. (2020). Delirium Detection Based on the Clinical Experience of Pediatric Intensivists. Pediatr. Crit. Care Med..

[95] Ista E., Redivo J., Kananur P., Choong K., Colleti J., Needham D.M., Awojoodu R., Kudchadkar S.R. (2022). ABCDEF Bundle Practices for Critically Ill Children: An International Survey of 161 PICUs in 18 Countries. Crit. Care Med..

[96] Kim K., Jeong J.H., Choi E.K. (2024). Non-pharmacological interventions for delirium in the pediatric population: A systematic review with narrative synthesis. BMC Pediatr..

[97] Stenkjaer R.L., Herling S.F., Egerod I., Weis J., van Dijk M., Kudchadkar S.R., Ramelet A.S., Ista E. (2022). Development of a non-pharmacologic delirium management bundle in paediatric intensive care units. Nurs. Crit. Care.

[98] Witte M.A., Lloyd R.M., McGree M., Kawai Y. (2023). Sleep quantity and quality of critically ill children perceived by caregivers and bedside nursing staff: A pilot study. J. Clin. Sleep. Med..

[99] Choong K., Fraser D.D., Al-Farsi A., Awlad Thani S., Cameron S., Clark H., Cuello C., Debigaré S., Ewusie J., Kennedy K. (2024). Early Rehabilitation in Critically ill Children: A Two Center Implementation Study. Pediatr. Crit. Care Med..

[100] Sosnowski K., Lin F., Chaboyer W., Ranse K., Heffernan A., Mitchell M. (2023). The effect of the ABCDE/ABCDEF bundle on delirium, functional outcomes, and quality of life in critically ill patients: A systematic review and meta-analysis. Int. J. Nurs. Stud..

[101] Simone S., Edwards S., Lardieri A., Walker L.K., Graciano A.L., Kishk O.A., Custer J.W. (2017). Implementation of an ICU Bundle: An Interprofessional Quality Improvement Project to Enhance Delirium Management and Monitor Delirium Prevalence in a Single PICU. Pediatr. Crit. Care Med..

[102] Lin J.C., Srivastava A., Malone S., Jennison S., Simino M., Traube C., LaRose K., Kawai Y., Neu L., Kudchadkar S. (2023). Caring for Critically Ill Children with the ICU Liberation Bundle (ABCDEF): Results of the Pediatric Collaborative. Pediatr. Crit. Care Med..

[103] Lewis K., Balas M.C., Stollings J.L., McNett M., Girard T.D., Chanques G., Kho M.E., Pandharipande P.P., Weinhouse G.L., Brummel N.E. (2025). A Focused Update to the Clinical Practice Guidelines for the Prevention and Management of Pain, Anxiety, Agitation/Sedation, Delirium, Immobility, and Sleep Disruption in Adult Patients in the ICU. Crit. Care Med..

[104] Flükiger J., Hollinger A., Speich B., Meier V., Tontsch J., Zehnder T., Siegemund M. (2018). Dexmedetomidine in prevention and treatment of postoperative and intensive care unit delirium: A systematic review and meta-analysis. Ann. Intensive Care.

[105] Serpa Neto A., Young M., Phongphithakchai A., Maeda A., Hikasa Y., Pattamin N., Kitisin N., Premaratne G., Chan G., Furler J. (2025). A target trial emulation of dexmedetomidine to treat agitation in the intensive care unit. Crit. Care Sci..

[106] Wu H., Wu P., Xiang L., Huang Q., Xiang Y., Zhang J., Zhao Z., Xu T. Effect of different intranasal dexmedetomidine doses on pediatric postoperative delirium and agitation: Network meta-analysis. Pediatr. Res..

[107] Tomlinson E.J., Schnitker L.M., Casey P.A. (2024). Exploring Antipsychotic Use for Delirium Management in Adults in Hospital, Sub-Acute Rehabilitation and Aged Care Settings: A Systematic Literature Review. Drugs Aging.

[108] Devlin J.W., Skrobik Y., Gélinas C., Needham D.M., Slooter A.J.C., Pandharipande P.P., Watson P.L., Weinhouse G.L., Nunnally M.E., Rochwerg B. (2018). Clinical Practice Guidelines for the Prevention and Management of Pain, Agitation/Sedation, Delirium, Immobility, and Sleep Disruption in Adult Patients in the ICU. Crit. Care Med..

[109] Liviskie C., McPherson C., Luecke C. (2021). Assessment and Management of Delirium in the Pediatric Intensive Care Unit: A Review. J. Pediatr. Intensive Care.

[110] Turkel S.B. (2017). Pediatric Delirium: Recognition, Management, and Outcome. Curr. Psychiatry Rep..

[111] Slooff V.D., van den Dungen D.K., van Beusekom B.S., Jessurun N., Ista E., Tibboel D., de Wildt S.N. (2018). Monitoring Haloperidol Plasma Concentration and Associated Adverse Events in Critically Ill Children with Delirium: First Results of a Clinical Protocol Aimed to Monitor Efficacy and Safety. Pediatr. Crit. Care Med..

[112] Turkel S.B., Hanft A. (2014). The pharmacologic management of delirium in children and adolescents. Paediatr. Drugs.

[113] Sassano-Higgins S., Freudenberg N., Jacobson J., Turkel S. (2013). Olanzapine reduces delirium symptoms in the critically ill pediatric patient. J. Pediatr. Intensive Care.

[114] Joyce C., Witcher R., Herrup E., Kaur S., Mendez-Rico E., Silver G., Greenwald B.M., Traube C. (2015). Evaluation of the Safety of Quetiapine in Treating Delirium in Critically Ill Children: A Retrospective Review. J. Child. Adolesc. Psychopharmacol..

[115] Groves A., Traube C., Silver G. (2016). Detection and Management of Delirium in the Neonatal Unit: A Case Series. Pediatrics.

[116] Kishk O.A., Simone S., Lardieri A.B., Graciano A.L., Tumulty J., Edwards S. (2019). Antipsychotic Treatment of Delirium in Critically Ill Children: A Retrospective Matched Cohort Study. J. Pediatr. Pharmacol. Ther..

[117] Capino A.C., Thomas A.N., Baylor S., Hughes K.M., Miller J.L., Johnson P.N. (2020). Antipsychotic Use in the Prevention and Treatment of Intensive Care Unit Delirium in Pediatric Patients. J. Pediatr. Pharmacol. Ther..

[118] Traube C., Witcher R., Mendez-Rico E., Silver G. (2013). Quetiapine as treatment for delirium in critically ill children: A case series. J. Pediatr. Intensive Care.

[119] Caballero A., Bashqoy F., Santos L., Herbsman J., Papadopoulos J., Saad A. (2023). Quetiapine for the Treatment of Pediatric Delirium. Ann. Pharmacother..

[120] Thielen J.R., Sawyer J.E., Henry B.M., Zebracki J., Cooper D.S., Koh W. (2024). Short-Term Effect of Quetiapine Used to Treat Delirium Symptoms on Opioid and Benzodiazepine Requirements in the Pediatric Cardiac Intensive Care Unit. Pediatr. Cardiol..

[121] Ruth O., Malas N. (2024). Neonatal delirium. Semin. Fetal Neonatal Med..

