# Correction: de Almeida et al. The Challenges of Diagnosing, Managing, and Preventing Pediatric Delirium. *Children* 2025, *12*, 918

**DOI:** 10.3390/children12081050

**Published:** 2025-08-11

**Authors:** Juliana Patrícia Chaves de Almeida, Yu Kawai, Arnaldo Prata-Barbosa, Roberta Esteves Vieira de Castro

**Affiliations:** 1 Pediatric Intensive Care Unit, Department of Pediatrics, Federal Hospital of Lagoa, Rio de Janeiro 22470-050, Brazil; juliana.almeida@souzamarques.br; 2 Department of Pediatrics, Souza Marques School of Medicine, Rio de Janeiro 21310-310, Brazil; 3 Department of Pediatrics, Division of Pediatric Critical Care Medicine, Mayo Clinic Children’s, Rochester, MN 55905, USA; kawai.yu@mayo.edu; 4 Department of Pediatrics, D’Or Institute of Teaching and Research (IDOR), Rio de Janeiro 22281-100, Brazil; arnaldo.prata@idor.org; 5 Department of Pediatrics, Pedro Ernesto University Hospital, Rio de Janeiro State University (UERJ), Rio de Janeiro 20550-013, Brazil

## Text Correction

In the published publication [1], there were three errors regarding the text. The original expression is not clear enough.

A correction has been made to “6.3. Cornell Assessment of Pediatric Delirium”, Paragraph 4:

“Assessments with the CAP-D can only be performed when the Richmond Agitation-Sedation Scale (RASS) score is between −3 and +4 or the State Behavioral Scale (SBS) is between −1 and +2 [62]. In screening for delirium in patients with developmental delay, if there was a RASS fluctuation of at least two points during a 24 h period along with a positive CAP-D, the specificity of diagnosis increased to 97%, the positive predictive value increased to 89%, and the negative predictive value remained similar at 87%, so this is superior than relying on the CAP-D alone [66]. The CAP-D is also available in multiple languages—Table 1”.

A correction has been made to “6.8. Which Tools Are Recommended Today?”, Paragraph 2:

“Another example is an international survey study conducted by Ista et al., which showed that only 44% of the 161 PICUs in North America, South America, and Europe routinely monitored for PD with a validated tool [95]. Finally, a study conducted in 28 United Kingdom and Ireland PICUs found that only 64% of the units implemented a PD screening protocol in 2023 despite large efforts for widespread PD education and training as well as building a national PD database [11]. These studies illustrate that, despite guidelines to routinely screen and diagnose PD to improve patient outcomes, buy-in from unit staff may be challenging, which reinforces the importance of frequent literature reviews and unit education among multidisciplinary staff”.

A correction has been made to “7.2. Pharmacological Approach”, Paragraph 6:

“Studies show that polypharmacy is an independent risk factor for delirium, and therefore, a daily review of each prescription with consequent reduction in medications has been suggested [108,109]”.

## References Correction

The replacement of references 66 and 95 appears below:

66. Kaur, S.; Silver, G.; Samuels, S.; Rosen, A.H.; Weiss, M.; Mauer, E.A.; Geber, L.M.; Greenwald, B.M.; Traube, C. Delirium and Developmental Disability: Improving Specificity of a Pediatric Delirium Screen. *Pediatr. Crit. Care Med*. **2020**, *21*, 409–414.
95. Ista, E.; Redivo, J.; Kananur, P.; Choong, K.; Colleti, J., Jr.; Needham, D.M.; Awojoodu, R.; Kudchadkar, S.R. ABCDEF Bundle Practices for Critically Ill Children: An International Survey of 161 PICUs in 18 Countries. *Crit. Care Med.* **2022**, *50*, 114–125.

The authors state that the scientific conclusions are unaffected. This correction was approved by the Academic Editor. The original publication has also been updated.
